# Aerosol‐Assisted Chemical Vapor Deposition of Mixed Phase Tin Phosphide Thin Films and Their Anode Performance for Sodium‐Ion Batteries

**DOI:** 10.1002/cssc.71205

**Published:** 2026-11-05

**Authors:** Kaiqing Fan, Xiang Lian, Kangping Ma, Veronica Celorrio, Geoffrey Hyett, Andrew L. Hector

**Affiliations:** ^1^ School of Chemistry and Chemical Engineering University of Southampton Southampton UK; ^2^ Diamond Light Source Harwell Science & Innovation Campus Didcot UK

**Keywords:** aerosol‐assisted chemical vapor deposition, sodium‐ion batteries, thin films, tin phosphides

## Abstract

SnP/Sn and SnP/Sn_4_P_3_ thin films were synthesized via aerosol‐assisted chemical vapor deposition on titanium foils. N‐phenyl phosphine carboxamide was used for the first time in chemical vapor deposition as an air‐stable phosphorus precursor, together with tin(IV) acetate in methanol or chloroform, respectively. The resulting films served as free‐standing electrodes in sodium half cells, eliminating the need for binders and conductive additives. Both types of films exhibited good electrochemical performance, delivering discharge capacities exceeding 500 mA h g^−1^ after 100 cycles at 200 mA g^−1^. Ex situ diffraction and X‐ray absorption spectroscopy revealed reversible and irreversible changes in SnP/Sn during cycling. The SnP phase becomes amorphous after the first reduction and remains largely amorphous in subsequent cycles, but X‐ray absorption spectroscopy provides evidence for (amorphous) SnP reformation during oxidation. The results provide direct structural evidence for a conversion–alloying mechanism and highlight the structural stability of amorphous Sn–P networks during repeated sodiation/desodiation.

## Graphical abstract

*N*‐phenylphosphinecarboxamide is an effective air‐stable phosphorus precursor to make tin phosphides by CVD. The thin films on metal foil served as electrodes in sodium half cells. The highest discharge capacities exceeded 500 mA h g^−1^ after 100 cycles at 200 mA g^−1^. SnP becomes amorphous on conversion to Sn and Na_3_P, but XAS provides evidence for SnP reformation during oxidation.

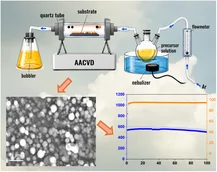

## 1 Introduction

Sodium‐ion batteries (SIBs) are a promising alternative to lithium‐ion batteries in many applications, driven by the earth abundance of sodium and hence its lower cost [1]. However, sodium has a larger ionic radius (0.102 nm) [2] and lower electrochemical activity, leading to poorer cycle stability and sluggish charging kinetics [3]. The commercial graphite anode for LIBs cannot be applied to sodium storage because of its small interlayer spacing [4]. Carbonaceous electrode materials for SIBs, such as “soft” and “hard” carbon, have limited capacity (up to ~350 mA h g^−1^) [5]. Group 14 and 15 elements such as phosphorus [6], antimony [7], and tin [8] have been extensively studied as anode materials because they have high capacity based on the formation of sodium‐rich alloys. However, large volume changes (e.g., 490% from P to Na_3_P) can cause rapid pulverization and degradation of the electrodes [9].

Tin phosphides (Sn_4_P_3_ [10], SnP [11, 12], and SnP_3_ [12]) undergo stepwise formation of Na_3_P and Na─Sn alloys in sodium cells (conversion and alloying reactions). Hence, they have high theoretical capacities (>1000 mA h g^−1^) [13], and the structural deterioration during cycling is partially mitigated by Na_3_P formation alongside alloy formation [14]. The presence of metallic tin also aids electronic conductivity. Qian et al. used a Sn_4_P_3_/carbon composite synthesized by high‐energy mechanical milling as a sodium battery anode [15]. The as‐prepared nanocomposite showed an initial discharge capacity of 850 mA h g^−1^ with 86% of the capacity retained over 150 cycles. Tang and coworkers incorporated tin monophosphide (SnP) nanocrystals into the interlayer spacing of the MXene Ti_3_C_2_T_*x*_ (T represents surface terminations) using a solvothermal method, enabling ultrafast kinetics of sodium storage [16]. The material exhibited excellent rate performance, with a reversible capacity of 438 mA h g^−1^ even at 15 A g^−1^.

Understanding the charge/discharge mechanism of tin phosphides in SIBs is critical if the electrochemical performance is to be further improved [17]. For Sn_4_P_3_, X‐ray diffraction (XRD) has given varied results. Kim et al. [18] and Xu et al. [19] combined ex situ XRD and X‐ray photoelectron spectroscopy to show that the Sn─P bond of Sn_4_P_3_ can be reformed to regenerate a low‐crystallinity material after cycling. There is only one study on the charge storage mechanism of SnP in sodium cells, which only reveals that the SnP phase is not seen after the first cycle [11, 12]. Tin compounds and their sodiation products often undergo amorphization during cycling, so techniques that do not rely on long‐range crystalline structure, such as XPS and X‐ray absorption spectroscopy (XAS), can be important in understanding their charge storage mechanisms.

Significant achievements have been made in tin phosphide‐based anode materials, but their preparation methods are still mainly limited to ball‐milling and solvothermal synthesis [19, 20]. Chemical vapor deposition (CVD) is an efficient method to prepare thin films with adjustable particle sizes, and some of these have provided effective anode materials. For example, Liao and coworkers deposited ZnSb on graphite substrates by atmospheric pressure CVD that exhibited a capacity of 225 mA h  g^−1^ at a current density of 750 mA  g^−1^ in SIBs [21]. Zhu et al. used aerosol‐assisted (AA) CVD, which has a lower precursor volatility requirement than conventional CVD, to grow tin(II) sulfide on stainless‐steel foil that offered enhanced SIB cycling performance compared with SnS materials obtained by other methods [22].

The classical CVD routes to metal phosphides, developed largely around the III‐V semiconductors, use PH_3_ as the phosphorus source [23]. P(SiMe_3_)_3_ was developed as an alternative to phosphine that is safer and easier to handle, although it also produces phosphine in moist conditions. Precursors such as t‐butyl or cyclohexyl phosphines have been successfully used to produce transition‐metal phosphide films [24], while PCl_3_ can also be used in conjunction with hydrogen [25]. In a recent breakthrough, zinc phosphide was prepared by AACVD using air‐stable, solid‐state phosphine carboxamide (PH_2_CONH_2_) as the phosphorus source and a simple acetate for the zinc source [26]. In contrast to the aforementioned phosphorus precursors, it can be stored under ambient conditions and is less flammable and corrosive.

Herein, we deposit tin phosphide films using *N*‐phenyl phosphine carboxamide (PH_2_CONHC_6_H_5_) and tin(IV) acetate by AACVD. *N*‐phenyl phosphine carboxamide is simple to synthesize in water and isolate. Depending on the solvent used, SnP/Sn or SnP/Sn_4_P_3_ mixtures were prepared. When deposited onto metal foil current collectors, these were applied as binder‐free anodes for SIBs. Both materials exhibited enhanced cycling and rate performances compared with previously reported pure‐phase tin phosphide materials and were comparable to some carbon‐based composites. This is ascribed to their small particle size and strong adhesion to the current collector, improving structural stability and ion transport kinetics. The tin oxidation states and phase behavior of the SnP/Sn films are investigated by ex situ XRD and XAS during sodiation and desodiation.

## 2 Results and Discussion

### 2.1 Synthesis and Characterization of *N*‐Phenyl Phosphine Carboxamide

Tin phosphide thin films were prepared by AACVD, in which PH_2_CONHC_6_H_5_ was selected as a novel phosphorus precursor. The synthesis of PH_2_CONHC_6_H_5_ is based on a nucleophilic addition reaction between aniline and the 2‐phosphaethynolate anion (PCO^−^), as illustrated in Figure 1. The resulting product, a white powder, was characterized by nuclear magnetic resonance (NMR) spectroscopy in chloroform‐D, and spectral results are represented in Figure S2. In the ^1^H NMR spectrum, a singlet appearing at 7.7 ppm is attributed to the N–H proton. The aromatic protons of the phenyl ring give rise to a set of multiplets at 7.67 (s), 7.40 (d), 7,24 (t), and 7.06 ppm (d). Additionally, two distinct peaks located at 3.5 and 4.0 ppm are ascribed to the P–H protons. In the ^13^C NMR spectrum, the carbonyl carbon is observed at 172.2 (^1^J_PC_ = 12.4 Hz) ppm and the aromatic carbons from 120.1 to 137.5 ppm. In the ^31^P NMR spectrum, a triplet centered at −123.7 ppm (^1^J_PH_ = 207.15 Hz) is indicative of P–H coupling. Duplicate combustion elemental analysis gave C 54.3/54.9% (54.9% expected), H 4.9/5.1% (5.3%), N 8.9/8.9% (9.1%), and P 20.4/20.3% (20.2%).

**FIGURE 1:**
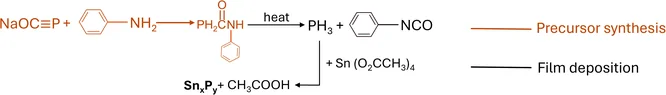
Schematic illustration of the synthesis of *N*‐phenyl phosphine carboxamide and its use in CVD of tin phosphides.

The phosphorus precursor used in this deposition is structurally similar to PH_2_CONH_2_, which has previously been used in CVD of zinc phosphide [26], but its extraction is more straightforward and can be achieved in an aqueous system. A phenyl group was introduced as a substituent as it facilitated easier separation and purification of the phosphorus precursor, making this a more scalable process. The growth mechanism of tin phosphides is presumed to follow Figure 1, with PH_2_CONHC_6_H_5_ generating PH_3_ in situ at high temperatures, which is immediately consumed for film deposition, avoiding the need to handle phosphines that are often highly toxic and pyrophoric.

### 2.2 Tin Phosphide Preparation and Characterization

Tin phosphides were first fabricated on glass (4 × 45 × 140 mm^3^) to test the feasibility of the growth of thin films. Using methanol as the solvent, depositions were carried out over a range of substrate temperatures (Table S1). At lower temperatures than 350 °C, no films were observed, and the resulting product became powdery with poor adhesion to the substrate at a higher temperature than 450 °C, This can be attributed to gas‐phase reactions. Within the accessible deposition window, films deposited at 350, 425, and 450 °C were identified as mixed phased copmpunds of SnP and Sn, with active‐material loadings of approximately 0.10, 0.15, and 0.18 mg, respectively. Among these samples, the film deposited at 425 °C showed the most favorable combination of film quality and electrochemical cycling behavior and was therefore selected for detailed investigation (denoted as SnP/Sn). XRD was employed to determine the phase composition of the samples, and the results are presented in Figure 2. XRD reveals a biphasic material composed of trigonal SnP (*P* $\bar{3}$ *m*1, ICSD 69026) and tetragonal Sn (*I*4_1_/*amd*, ICSD 252800). Rietveld refinement was performed to evaluate crystallite sizes and the relative amounts of coexisting phases. Parameters such as background, lattice constants, and scale factors were refined. The lattice constants of SnP (Table 1) were determined to be *a* = 4.37 Å and *c* = 6.05 Å (literature values *a* = 4.39 Å, *c* = 6.04 Å for SnP [27]). The refined phase ratio for SnP/Sn was 81 mol% SnP and 19 mol% elemental Sn (R_wp_ = 5.27%, Figure S3).

**FIGURE 2:**
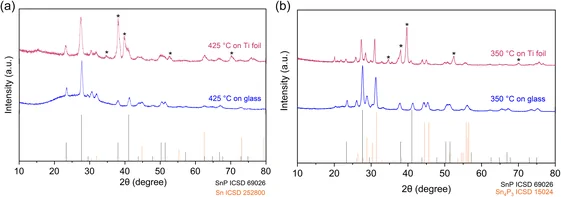
XRD patterns of the films deposited using (a) methanol or (b) chloroform solvent on glass and Ti foils. (Note: Peaks marked with * correspond to the background signals from the Ti substrate.).

**TABLE 1:** Summary of the analysis results of the films obtained under different conditions.

Sample	Solvent	Temperature, °C	phase	Lattice parameters		Crystallite size, nm	Sn/P ratio
				*a*, Å	*c*, Å		
SnP/Sn	Methanol	425	SnP	4.373(8)	6.053(12)	18	1.31:1
			Sn	5.833(16)	3.186(11)	43	
SnP/Sn_4_P_3_	Chloroform	350	SnP	4.370(24)	6.123(24)	72	1.13:1
			Sn_4_P_3_	3.999(17)	35.325(8)	276	

Using chloroform as the solvent requires a deposition temperature lower than 400 °C due to its tendency to decompose at higher temperatures [28]. Films deposited at 300, 350, and 400 °C exhibited distinct phase compositions, corresponding to Sn_4_P_3_, SnP/Sn_4_P_3,_ and SnP/Sn_4_P_3_, respectively, with active‐material loadings of approximately 0.08, 0.10, and 0.10 mg. Among these samples, the film deposited at 350 °C showed the most stable electrochemical behavior and the highest reversible capacity after prolonged cycling. This sample was therefore selected for detailed structural and electrochemical investigation and is hereafter denoted SnP/Sn_4_P_3_. Its diffraction pattern can be fitted with trigonal SnP (*P* $\bar{3}$ *m*1) and trigonal Sn_4_P_3_ (R‐3m:H, ICSD 15014), with lattice parameters of SnP at *a* = 4.37 Å and *c* = 6.12 Å, and Sn_4_P_3_ at *a* = 4.00 Å and *c* = 35.33 Å (literature values *a* = 3.97 Å, *c* = 35.33 Å for Sn_4_P_3_ [29]). The phase ratio was 54 wt% SnP and 46 wt% Sn_4_P_3_ (R_wp_ = 8.16%, Figure S3). Refined parameters are shown in Table 1. In both materials, the unit cell dimensions of SnP are very similar to the values in ICSD. The SnP/Sn films are rougher and can be scratched by tweezers, while SnP/Sn_4_P_3_ is smoother and more firmly attached to the substrate, showing greater resistance to surface damage. The differences in the evaporation behavior of the two solvents, methanol and chloroform, may play a crucial role in determining the morphology and phase composition of the films. The higher vapor pressure (21.9 vs. 12.9 kPa at 20 °C) and slightly lower boiling point (61 vs. 65 °C) of chloroform compared to methanol will affect the evaporation behavior of aerosol droplets. Rapid solvent loss during aerosol transport promotes nucleation and particle formation, leading to the observed spherical morphology. This kinetically controlled growth process may limit phase separation or metallic Sn formation, favoring the direct formation of tin phosphide phases.

To obtain binder‐free electrodes, the films were grown onto a metal foil current collector. Titanium foil was chosen because of its good electrical conductivity, compatibility for the XAS measurements, and resistance to alloy formation with tin. Using the conditions developed through deposition on glass, the films on titanium exhibited similar diffraction patterns and compositions to those grown on glass (Figure 2). The morphologies of the films on Ti foils are shown in Figure 3. SnP/Sn has a uniform film mainly containing plate‐like particles (550 × 400 × 30 nm^3^), reflecting the crystal structure, and a small amount of spherical particles with a diameter of around 300 nm. SnP/Sn_4_P_3_ obtained from chloroform is a silver‐gray film consisting of microspheres with diameters ranging from 100–450 nm. The lighter‐colored spherical particles in both images are likely formed because of some gas‐phase reaction. The particle morphologies of films deposited on glass are similar (Figure S4). Energy‐dispersive X‐ray spectroscopy (EDS) mapping shows that Sn and P are uniformly distributed on the Ti foil for both films (Figure 3 for SnP/Sn and Fig. S4 for SnP/Sn_4_P_3_). The EDS shows the Sn content of SnP/Sn and SnP/Sn_4_P_3_ to be 56% and 53%, respectively, which are in agreement with the XRD phase analysis.

**FIGURE 3:**
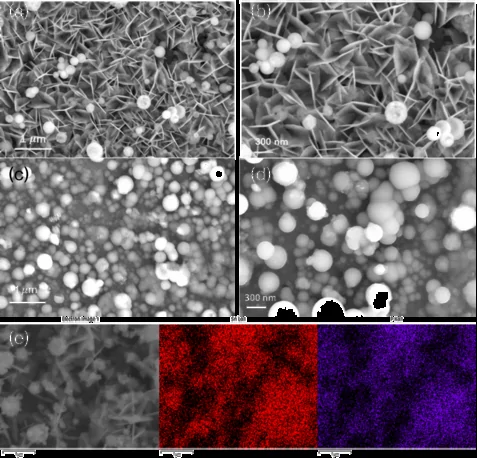
SEM images of (a,b) SnP/Sn and (c,d) SnP/Sn_4_P_3_ at different magnifications. (e) EDX element mapping of Sn (red) and P (purple) for SnP/Sn—a similar map for SnP/Sn_4_P_3_ can be found in Figure S5.

### 2.3 Sodium Half Cells

Cyclic voltammograms of SnP/Sn and SnP/Sn_4_P_3_ in sodium half‐cells are shown in Figure 4. In SnP/Sn, a cathodic peak is observed at 1.00 V in the initial scan and 1.09 V subsequently. A peak at 0.19 V in that first reduction cycle is not observed in subsequent cycles, a sign that it may be due to the formation of a solid electrolyte interphase (SEI) layer. A less intense, broad reduction peak is also observed at 0.38–0.55 V. In the anodic scans, there are peaks located at 0.39 and 0.70 V. From the second cycle, the curves overlapped well, demonstrating good stability of the electrochemical processes. SnP/Sn_4_P_3_ has similar features, also exhibiting SEI formation behavior in the first cycle and then stable cycling afterward. Compared with SnP/Sn, the cyclic voltammetry (CV) curve of Sn_4_P_3_/SnP shows a pronounced shift in the first oxidation peak to lower voltage of 0.26 V, which may be associated with the Sn_4_P_3_ phase [11, 12]. The CV features of SnP/Sn are free from interference by the Sn_4_P_3_ phase, and SnP/Sn contains more than 80 mol% SnP, making it a good candidate for mechanistic investigations of the SnP charge storage mechanism.

**FIGURE 4:**
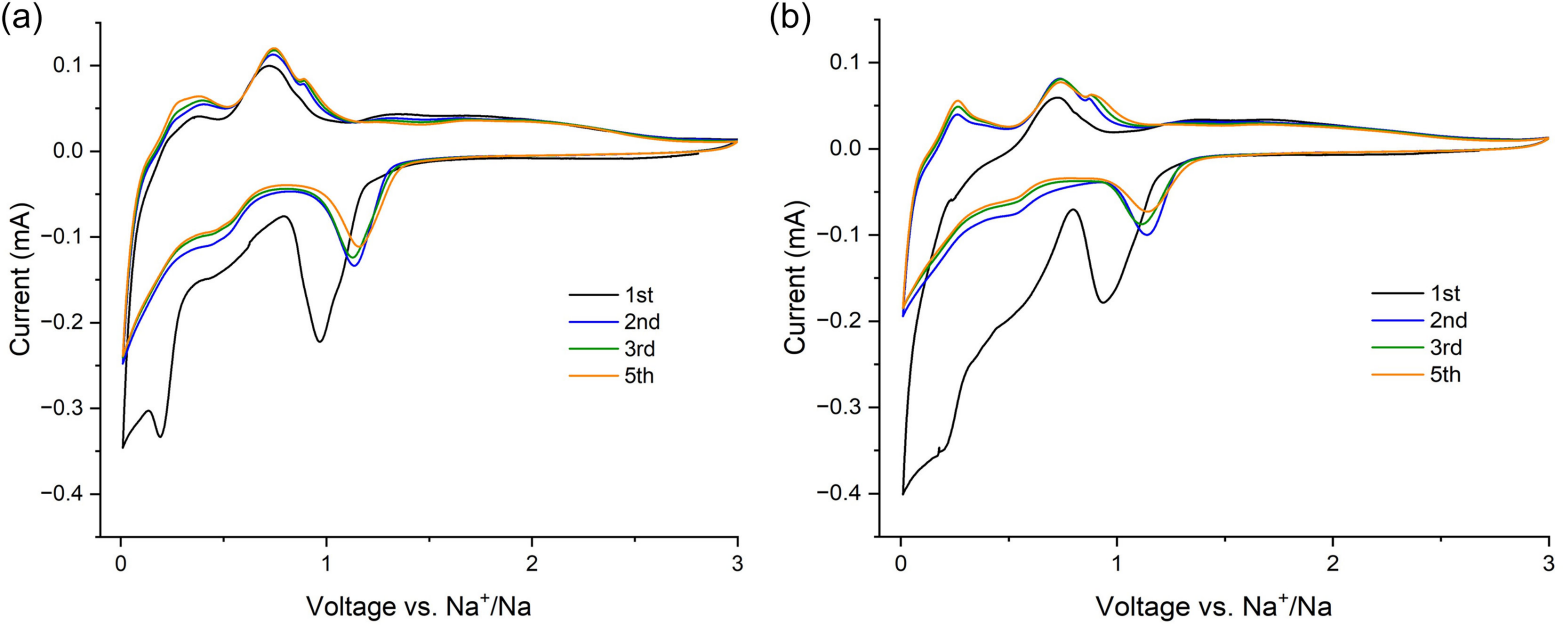
CV profiles of (a) SnP/Sn and (b) SnP/Sn_4_P_3_ at a scan rate of 1 mV s^−1^ with a potential range of 0.01–3.0 V versus Na^+^/Na.

The sodium storage behavior of the tin phosphides was further investigated under galvanostatic conditions (200 mA g^−1^) in the voltage range of 0.005–3 V (vs. Na^+^/Na). In the charge/discharge curves displayed in Figure 5, SnP/Sn has a reduction/oxidation specific capacity of 1153/747 mA h g^−1^ with an initial coulombic efficiency (ICE) of 65% in the first cycle. It is notable that much of this capacity loss is associated with the feature of around 1.2 V in the reduction curve. In the subsequent cycles, the curves tend to overlap, with discharge plateaus observed at 1.30 and 0.25 V and a charge plateau at 0.68 V, similar potentials to those seen in the CV data.

**FIGURE 5:**
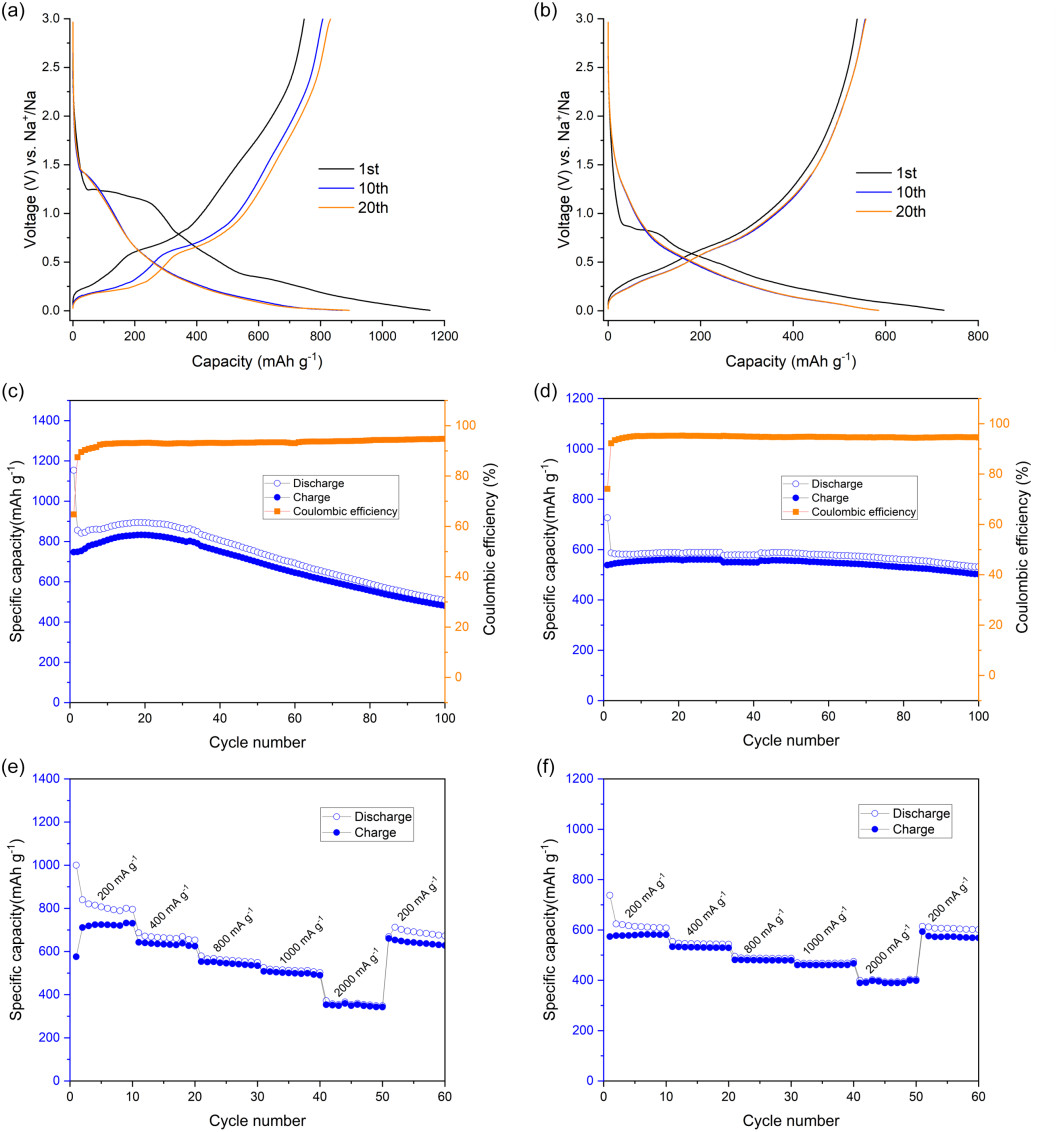
Electrochemical performance of tin phosphide electrodes. (a,b) Galvanostatic charge/discharge plots for the 1st, 10th, and 20th cycles (0.005–3 V versus Na^+^/Na at 200 mA g^−1^), (c,d) Capacity and coulombic efficiencies at 200 mA g^−1^, and (e,f) rate capabilities under various current densities of (a,c,e) SnP/Sn and (b,d,f) SnP/Sn_4_P_3_ electrodes.

Comparatively, SnP/Sn_4_P_3_ has lower initial reduction and oxidation capacities of 726 and 538 mA h g^−1^ but a higher ICE of 74%. This may be ascribed to its smaller surface area and fewer exposed active sites caused by larger particle size, leading to less capacity associated with irreversible reactions. Reduction and oxidation potential plateaus were identified at 0.24 and 0.64 V. The cycling performances of the two selected films are compared in Figure 5, while additional electrodes prepared at neighboring deposition temperatures are shown in Figure S6. For the methanol‐derived SnP/Sn series, the electrode deposited at 425 °C exhibited the most stable long‐term cycling behavior. After reaching a reduction capacity of 856 mA h g^−1^ in the second cycle, SnP/Sn‐425 retained 507 mA h g^−1^ after 100 cycles at 200 mA g^−1^, corresponding to 60% capacity retention. In comparison, SnP/Sn‐350 showed a substantially lower reversible capacity and underwent short circuit after 24 cycles, while SnP/Sn‐450 retained a lower capacity. The chloroform‐derived SnP/Sn_4_P_3_ showed superior cycling stability, retaining 531 mA h g^−1^ after 100 cycles, corresponding to 91% of its second‐cycle capacity. The phase‐pure Sn_4_P_3_ electrode deposited at 300 °C delivered a lower reversible capacity, retaining 378 mA h g^−1^ after 100 cycles, whereas the 400 °C SnP/Sn_4_P_3_ electrode showed pronounced cycle‐to‐cycle fluctuations in both capacity and Coulombic efficiency before the cycling test terminated after approximately 62 cycles. Although the loading does not vary independently from other deposition‐dependent film characteristics, the differences of capacities observed for the higher or lower loading electrode suggest that mass loading may contribute to the observed cycling behavior. To further assess cycling stability, both films were also cycled at 600 mA g^−1^ for 150 cycles, showing stable Coulombic efficiencies with gradual capacity fading (Figure S7).

As shown in Figure 5f, SnP/Sn and SnP/Sn_4_P_3_ electrodes were each cycled at various current densities to evaluate their rate capabilities. SnP/Sn possesses reversible capacities of 807, 664, 560, 513, and 354 mA h g^−1^ at current densities of 200, 400, 800, 1000, and 2000 mA g^−1^. During the initial cycles, the sample exhibits lower CE, which may be because of the enhanced reactions between the active material and electrolyte at low rate. In comparison, SnP/Sn_4_P_3_ shows better performance, with capacities of 614, 545, 487, 467, and 393 mA h g^−1^, respectively. This could be due to its strong adhesion to the current collector and the enhanced electronic conductivity from Sn_4_P_3_.

The binder‐free tin phosphide electrodes obtained via AACVD have significantly improved capacity and better cycling performance in sodium half‐cells compared with previously reported pure Sn_4_P_3_ materials. Moreover, their electrochemical behavior is also comparable to that of multiple tin phosphide/carbon composites (Table 2). This could be explained by the adhesion of the films to the current collectors, suggesting strong electronic contact and a relatively small particle size that may control the impact of volume expansion and facilitates the kinetics of Na‐ion transport.

**TABLE 2:** Performance of some tin phosphides and tin phosphides/C composites in SIBs.

Material	Initial discharge capacity, mA h g^−1^	Final discharge capacity, mA h g^−1^	Current density, mA g^−1^	Number of cycles	Refs.
SnP nanocrystals	850	600	100	200	[11, 12]
Sn_4_P_3_ nanoparticles	1200	305	50	10	[30]
Sn_4_P_3_	~900	~600	100	100	[18]
Sn_*x*_P_*y*_/C yolk–shell nanostructure	1252	442	500	100	[31]
Sn_4_P_3_/PN‐doped C	1066	398	100	100	[32]
Sn_4_P_3_/rGO	2138	510	200	100	[33]
Yolk–shell Sn_4_P_3_/C	—	515/360	100/1500	50/400	[34]
SnP/Sn	1153	507	200	100	This work
SnP/Sn_4_P_3_	726	531	200	100	This work

### 2.4 Postcycling Morphology and Impedance Analysis

The Nyquist plots of the two electrode types exhibit distinct impedance evolution during cycling (Figure 6). For the SnP/Sn electrode, the diameter of the semicircle decreases between the 5th and 50th cycles, indicating a reduction in charge‐transfer resistance (*R*_ct_). This decrease may be associated with progressive electrochemical activation of the electrode during cycling. Upon further cycling, however, the semicircle increases in size, indicating an increase in charge‐transfer resistance. Such an increase may be associated with the structural evolution of the electrode during repeated conversion–alloying reactions, including the possible aggregation of metallic Sn. Detailed fitting of the high‐frequency region of the electrochemical impedance spectra (EIS) spectra and the corresponding quantitative results are provided in Figure S10 and Table S3. Consistent with the changes observed by EIS, the postcycling SEM image of the SnP/Sn electrode after 100 cycles (Figure 6) reveals pronounced surface cracking, indicating substantial morphological degradation during cycling. In contrast, the SnP/Sn_4_P_3_ electrode exhibits smaller changes in impedance during cycling, suggesting more stable charge‐transfer behavior. The postcycling SEM image also shows that the surface morphology of the SnP/Sn_4_P_3_ electrode is comparatively well preserved, with fewer pronounced cracks than those observed for the SnP/Sn electrode.

**FIGURE 6:**
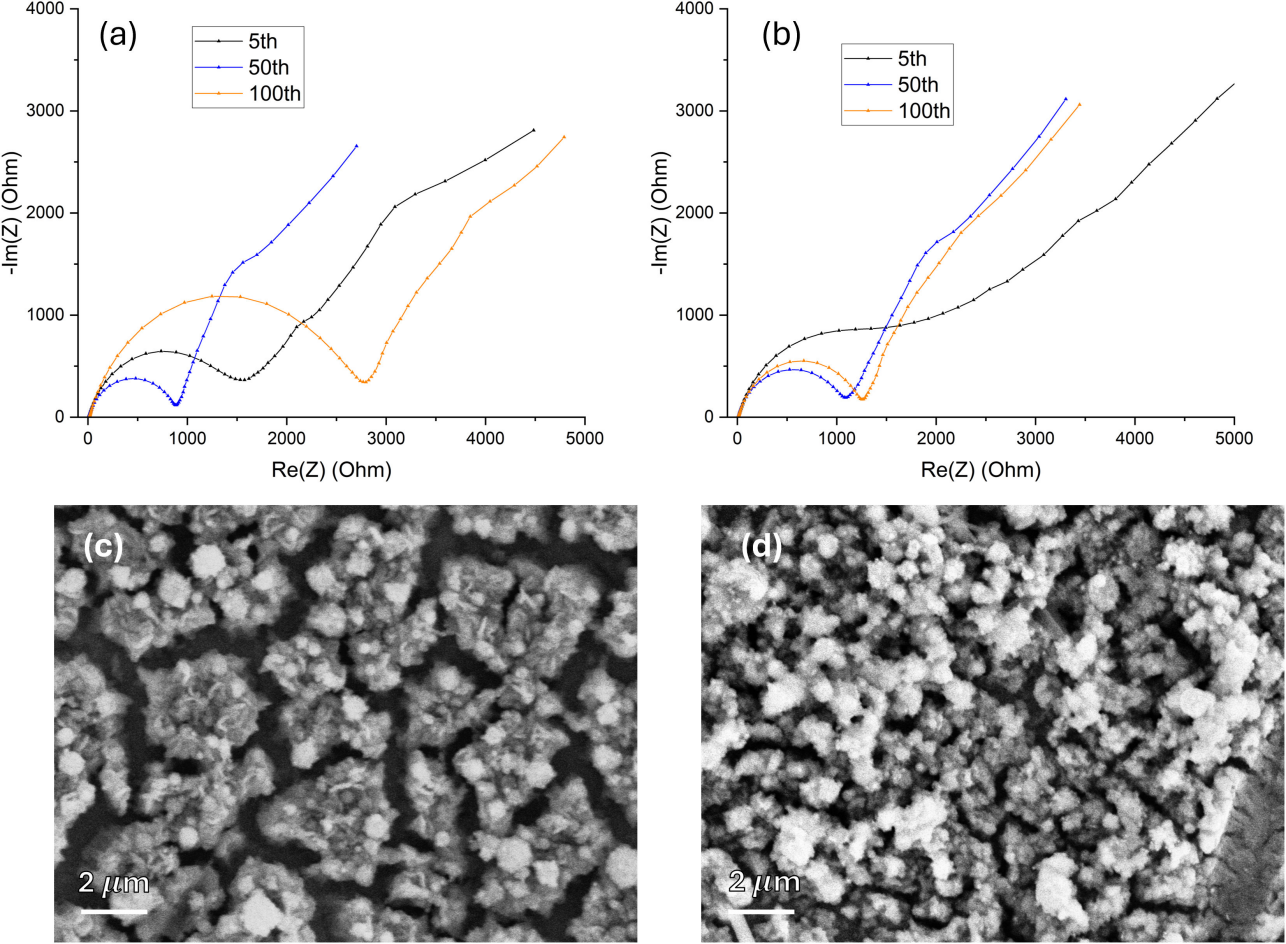
The Nyquist plots of (a) SnP/Sn and (b) SnP/Sn_4_P_3_ at 5th, 50th, and 100th cycles. SEM images of (c) SnP/Sn and (d) SnP/Sn_4_P_3_ after 100 charge–discharge cycles.

### 2.5 Sodium Storage Mechanism of SnP/Sn Anodes

Mechanistic investigations of the sodium battery behavior of tin phosphide anodes have primarily focused on Sn_4_P_3,_ while the (de)sodiation behavior of the metastable SnP phase is mainly unexplored. Previous reports on multiphase Sn–P electrodes have suggested that phase coexistence can provide beneficial kinetic and pseudocapacitive effects [35], which may contribute to the improved cycling stability observed for the SnP/Sn_4_P_3_ electrode. However, the substantial overlap of the redox processes associated with SnP and Sn_4_P_3_ makes direct assignment of the individual phase transformations more complex in the mixed‐phase electrode. Hence, the sodium storage mechanism of SnP in the SnP‐rich SnP/Sn film was examined to elucidate changes that occur during redox processes and understand its associated degradation behavior.

Based on the charge/discharge curves (Figure 7), we prepared samples at several distinct states of reduction or oxidation, corresponding to different stages of the sodiation/desodiation process. The selected points are near the potential plateaus, indicating the electrode’s stepwise phase transitions. For the SnP/Sn electrode, the reduction peak observed around 1.1 V is not reported during reduction of Sn_4_P_3_ electrodes (remembering that SnP has not previously been studied) and appears to be mostly related to the capacity loss observed in the first cycle and hence may be due to interface formation. The reduction peak observed in the range of 0.38–0.55 V may be attributed to the formation of Na_3_P and Na_15_Sn_4_. The oxidation peaks at approximately 0.39 and 0.70 V have been assigned by Liu et al. [11, 12] to the dealloying of Na_15_Sn_4_ and the extraction of Na from Na_3_P, based on extrapolation from similar behavior by Sn_4_P_3_ [15, 33, 36].

**FIGURE 7:**
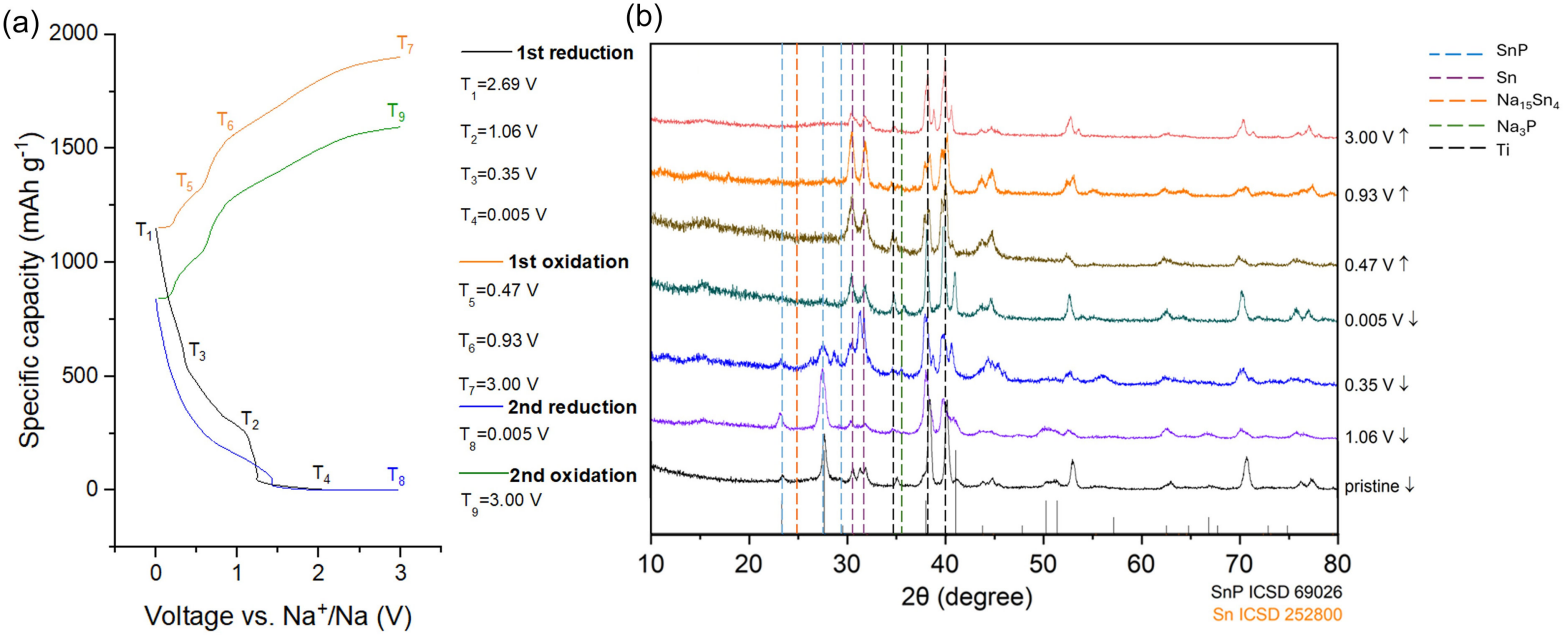
(a) Galvanostatic charge/discharge curves of SnP/Sn during the first two cycles at 200 mA g^−1^, and selected points for ex situ XRD and XAS measurements. (b) Ex situ XRD patterns of SnP/Sn during the first charge/discharge cycle.

The ex situ XRD patterns of SnP/Sn during the first cycle are shown in Figure 7. During reduction, no significant phase transition was observed during the voltage drop from open‐circuit voltage (OCV) to 1.06 V versus Na^+^/Na, indicating that this process mainly involves electrolyte decomposition or the insertion of Na^+^ into the electrode layers. When reduced to 0.35 V, the intensity of the SnP peaks significantly decreases, while that of metallic tin peaks increases, suggesting a conversion process dominates in this potential range. A newly appearing peak at about 35° 2θ is associated with the generation of Na_3_P. As the voltage reaches 0.005 V, the SnP peaks completely disappear, with a weak peak representing Na_15_Sn_4_ emerging at around 25°, showing that alloying reactions are taking place. Thus, XRD suggests a simple conversion and alloying process as illustrated by equations (1) and (2).

(1) $$\mathrm{SnP}+3{\mathrm{Na}}^{+}+3e^{‐}\;\to \;\mathrm{Sn}+{\mathrm{Na}}_{3}P$$

(2) $$4\mathrm{Sn}+15{\mathrm{Na}}^{+}+{15e}^{‐}\;\to \;{\mathrm{Na}}_{15}{\mathrm{Sn}}_{4}$$

On reoxidation from 0.005 to 0.93 V, the growth of tin peaks is observed, revealing a dealloying reaction. Upon further charging to 3 V, these peaks weaken to a height comparable to that of the pristine electrode. This implies that metallic tin may have been consumed through reformation of chemical bonds with phosphorus or other side reactions. However, the diffraction peaks of the SnP phase do not reappear, suggesting that this crystalline phase is not reversibly regenerated.

From the X‐ray absorption near‐edge structure (XANES) spectrum at the Sn K‐edge (Figure 8), during reduction, the height of the white line peak continuously decreases, approaching that of the Sn reference (at 0.35 V versus Na^+^/Na) at 0.3 V and dropping even further upon full sodiation. The decrease in white‐line peak intensity typically indicates a reduction in the oxidation state of tin, based on the previous findings reported by Yu et al. [37] These observations suggest that the valence state of tin in SnP declines, consistent with the transformation of SnP into Sn (Equation (1)), and subsequently becomes even lower, indicating Sn─Na alloy formation (Equation (2)). During desodiation, the white line peak intensity rises to a level higher than that of Sn reference when fully oxidized (3.0 V versus Na^+^/Na).

**FIGURE 8:**
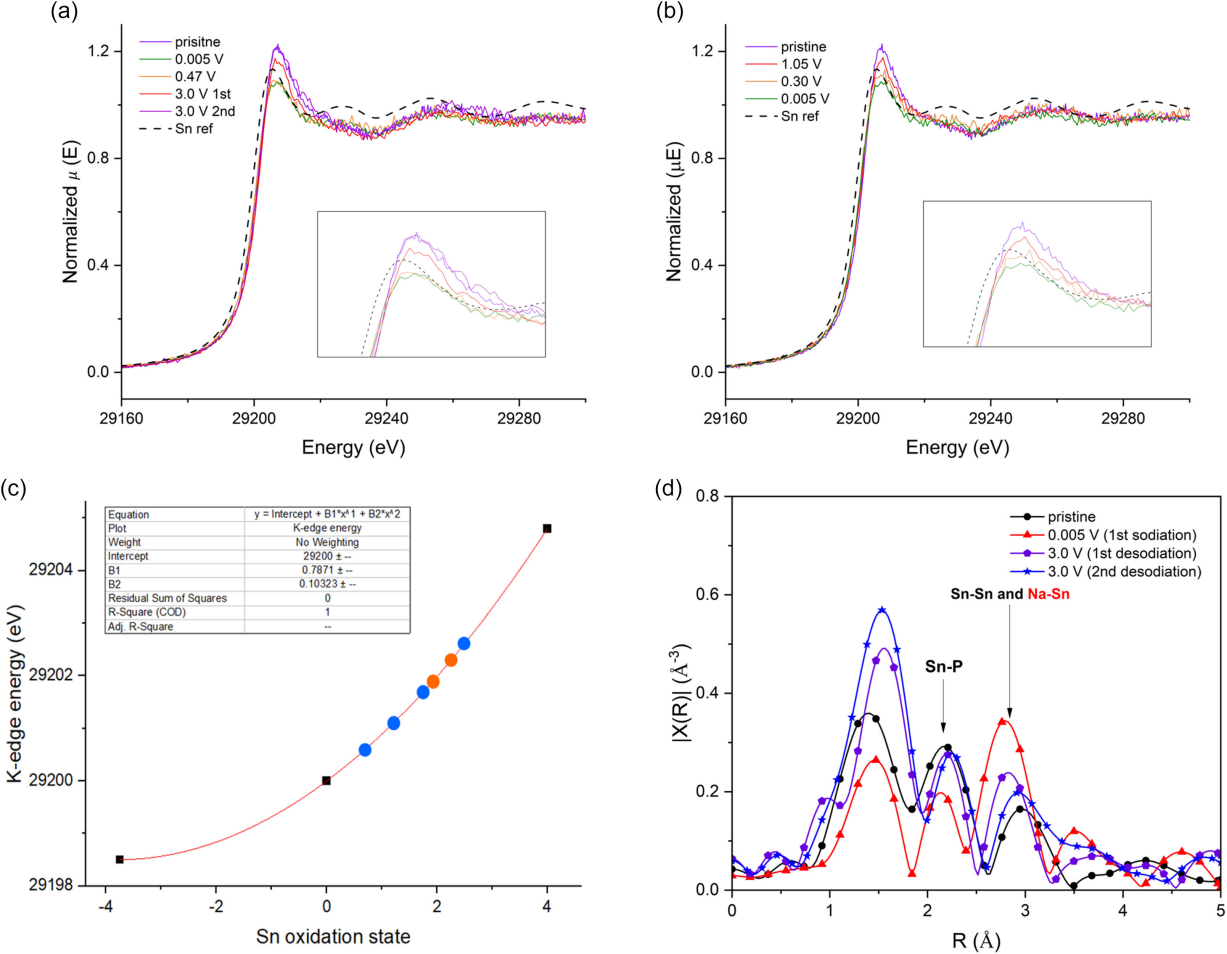
Sn K‐edge XANES spectra during (a) the first reduction and (b) the first oxidation process, including the final state after the second oxidation. (c) Oxidation states fitted with Sn K‐edge energies. (d) | *χ*(R)| profiles of ex situ EXAFS signals of SnP/Sn at OCV, first reduced, first reoxidized, and second reduced states.

Notably, after the second oxidation, the XANES spectrum closely overlaps with the pattern of the pristine electrode, which could imply that the Sn─P bonding is reformed to some extent. The edge positions measured from the maximum in the differential plots (Figure 7) do show a slightly different oxidation state trend to that seen in the white line intensities in that the average oxidation state appears to remain above that of the elemental tin. Using standards of SnO_2_ (+4), Sn (0), and Na_15_Sn_4_ (−3.75), a polynomial fitting method was employed to roughly estimate the oxidation states of Sn in the electrode at different stages of charge, as shown in Figure 8 and Table S2. It is worth noticing that the compositions of electrodes at various stages are complex and involve multiple phases; XANES provides an averaged measurement of the oxidation state of Sn. During the reduction‐oxidation process, the average oxidation state of tin in SnP/Sn drops from +2.49 to +0.70 and then grows back to a level (+2.25) slightly below that of the pristine material.

Figure 8 shows the |*χ*(R)| plots of the Sn K‐edge extended X‐ray absorption fine structure (EXAFS) data of SnP/Sn at different reduction and oxidation stages during 1st and 2nd cycles. The plot for the pristine material contains the features expected for Sn─P, with peaks around 2 to 2.5 Å close to the bonded Sn─P path, between 2.5 and 3 Å close to the first‐shell Sn─Sn path, and in the 3 to 4 Å range corresponding to the outer‐shell Sn─Sn paths. A further feature between 1 and 2 Å may indicate a Sn─O contribution from surface oxidation, although no tin oxides were observed by XRD. After the first reduction, the Sn─P signal weakens, accompanied by a dramatic increase in first‐shell Sn─Sn signal. In addition, a rightward shift in the Sn─Sn signal is observed, resulting from the appearance of a new Na─Sn path that overlaps with the Sn─Sn one. These features are consistent with the conversion and alloying mechanism suggested by the XRD. After oxidation, the intensity of the Sn─Sn peak decreases and its energy shifts to lower values; this observation is consistent with the dealloying of Na_15_Sn_4_ and the reoxidation of Sn into amorphous SnP. Concurrently, the peak corresponding to the Sn─P path increased to a height approaching that of the pristine material. Notably, the Sn─Sn peak, though diminished, remained more intense than in the pristine electrode, suggesting that a proportion of the metallic tin did not fully participate in the reversible formation of SnP. Qualitative P K‐edge EXAFS analysis indicates substantial disruption of P–Sn coordination during reduction, followed by partial recovery during reoxidation, while the P–P local environment also evolves during cycling (Figure S9).

The detailed EXAFS fitting results and the calculated interatomic distances on Sn K‐edge are summarized in Figure S8 and Table 3. As shown in Figure S8, the Sn─P peak is significantly weakened after full reduction, while the most intense peak can be better fitted by including both the Na─Sn path from Na_15_Sn_4_ and the Sn─Sn path from metallic tin, yielding an R‐factor of 4.2% compared with 5.6% when fitted using the Sn─Sn path alone. This provides evidence supporting the formation of Na─Sn alloys during the reduction process.

**TABLE 3:** EXAFS modeling results of the electrode after the completion of the sodiation or desodiation process.

State	Path	R, Å	*δ* ^2^	R‍‐‍factor, %
Pristine	Sn─P	2.66 ± 0.02	0.007 ± 0.001	1.3
	Sn─Sn	3.11 ± 0.03	0.007 ± 0.001	
	Sn─O	2.09 ± 0.02	0.007 ± 0.001	
1st sodiation (0.005 V)	Na─Sn	3.32 ± 0.03	0.009 ± 0.002	4.2
	Sn─Sn	2.96 ± 0.03	0.009 ± 0.002	
	Sn─P	2.59 ± 0.03	0.009 ± 0.002	
	Sn─O	2.03 ± 0.03	0.089 ± 0.002	
1st desodiation (3.0 V)	Sn─P	2.61 ± 0.04	0.010 ± 0.002	1.6
	Sn─Sn	3.03 ± 0.03	0.010 ± 0.002	
	Sn─O	2.08 ± 0.02	0.003 ± 0.004	
2nd desodiation (3.0 V)	Sn─P	2.63 ± 0.05	0.005 ± 0.003	2.7
	Sn─Sn	3.02 ± 0.04	0.005 ± 0.003	
	Sn─O	2.10 ± 0.02	0.005 ± 0.003	

In the pristine state, the Sn─P and Sn─Sn bond lengths are 2.66 and 3.11 Å, respectively, corresponding to a SnP‐type coordination environment. Upon the first reduction to 0.005 V, the Sn─P bond shortens to 2.59 Å, while new Sn–Sn contributions with shorter distances (~2.9 Å) appear, indicating partial cleavage of Sn─P bonds and the formation of metallic Sn clusters upon Na insertion. During the first reoxidation to 3.0 V, the Sn─P bond partially recovers to 2.61 Å, and the Sn–Sn distance slightly increases to 3.03 Å, suggesting that Sn─P bonds are reformed, although a fraction of metallic Sn remains unreacted. After the second reduction, the Sn─P bond length stabilizes around 2.63 Å, and the Sn─Sn signal persists (~3.02 Å), implying that the electrode reaches a quasi‐reversible configuration in which metallic Sn (originating from residual Sn‐rich domains and incomplete recombination after Na–Sn dealloying) and Sn–P species coexist. A similar conclusion was reached for Sn_4_P_3_ by Ding et al. [38], who attributed the shorter Sn─Sn path to the formation of small metallic Sn clusters. The evolution in the relative fractions of different scattering paths further corroborates this reaction process. The Sn–P fraction decreases markedly from 0.47 to 0.09 after the first reduction, accompanied by an increase in Sn–Sn from 0.26 to 0.70, indicating the formation of metallic Sn. Upon reoxidation, the Sn─P fraction partially recovers, while Sn─Sn decreases, suggesting reversible bond reconstruction. The Sn─O contribution remains nearly constant, likely originating from surface oxidation rather than bulk reactions.

Taken together, the XANES and EXAFS results demonstrate that during the reduction process, elemental Sn is extracted from SnP and subsequently alloys with Na to form Na_15_Sn_4_. Upon oxidation, Na_15_Sn_4_ undergoes dealloying, and a considerable proportion of the Sn rebonds with P to form amorphous SnP. However, once reduced, the crystalline SnP phase does not reappear in subsequent cycles, indicating an irreversible structural transformation. The electrochemically inactive Sn and the aggregation of Sn clusters (from the tin‐rich phase in the pristine material and the Sn released from the dealloying of Na_15_Sn_4_) during cycling may induce volume expansion and structural instability, contributing to the degradation of the SnP/Sn electrode upon prolonged cycling.

## 3 Conclusion

N‐phenyl phosphine carboxamide is an effective precursor to produce tin phosphides by AACVD while working with a simple tin acetate precursor and being safe and easy to handle. Composition of the depositions varied depending on whether methanol or dichloromethane was used as the carrier solvent. The SnP/Sn thin film mainly consists of SnP nanoflakes with some elemental tin, while the SnP/Sn_4_P_3_ thin film contained micro‐ and nano‐spheres (100–450 nm). When employed as binder‐free anodes in sodium half‐cells, both films exhibited very good cycling performance and rate capability, ascribed to their small particle sizes and strong adhesion to the current collector, which enhance structural integrity and both electron and ion transport. The sodium storage mechanism of SnP/Sn was explored by ex situ XRD and XAS. After the first cycle, a significant fraction of the SnP appears to participate in subsequent reversible reactions, albeit in an amorphous state, while the remaining Sn is retained in a metallic form and cycles independently via alloying/dealloying processes. This behavior may be associated with material degradation and capacity fading, providing implications for the future improvement of metal phosphide electrodes. Both film types deliver high gravimetric capacities, with more than 500 mA h g^−1^ maintained after 100 cycles at 200 mA g^−1^ and 600 mA g^−1^. The low loadings of these films of around 1.5 mg cm^−2^ mean the areal capacities are not high, around 0.75 mA h cm^−2^. However, CVD is highly applicable to deposition on structured substrates such as foams and nanocarbons, so the method could be used in future to also deliver high areal capacity.

## 4 Experimental Section

### 4.1 Synthesis of the N‐Phenyl Phosphine Carboxamide Precursor

NaOCP (dioxane)_0.37_ was synthesized according to a literature procedure [39], and the preparation of PH_2_CONHC_6_H_5_ was modified from a method used previously [40]. Specifically, 1.8 g of NaOCP (dioxane)0.37 (15.6 mmol), 1.27 g of aniline (13.7 mmol, 99.5%, Fisher), and 2.38 g of NaHCO_3_ (28.3 mmol) were added to 75 mL of deionized water in a round‐bottom flask. The mixture was stirred at room temperature overnight, and then the white powdered product was collected over a G4 glass frit, washed with deionized water, and quickly dried in vacuo.

### 4.2 Deposition of Thin Films by AACVD

SnP/Sn and SnP/Sn_4_P_3_ were both synthesized by AACVD from PH_2_CONHC_6_H_5_ and tin(IV) acetate (98%, Fisher) precursors. The glassware was washed with deionized water and acetone and then dried in an oven at 75 °C. For the growth of SnP/Sn, 0.10 g of PH_2_CONHC_6_H_5_ (6.53 × 10^−4^ mol) and 0.077 g of tin (IV) acetate (2.17 × 10^−4^ mol) were dissolved in 15 mL of anhydrous methanol. The precursor solution was added to a three‐neck flask, which was connected to an Ar gas source, a flowmeter, an AACVD reactor (design adapted from an earlier work) [41], and a bubbler (Figure S1). The substrates were titanium foil (0.01 × 24 × 120 mm^3^, 99.9%, Haiyuan Co.) and glass (145 × 45 × 4 mm^3^, SiO_2_‐coated float glass, Pilkington Ltd.). Before use, they were cleaned with acetone and placed on the carbon block in the reactor (initial attempts were carried out only on glass). The reactor was heated to 425 °C under flowing Ar. Once the temperature was stable, the precursor was aerosolized with a nebulizer and transported to the reactor with the Ar carrier gas (0.2 L min^−1^). The deposition process lasted for about 50 mins before the reactor was allowed to cool to room temperature. Additional methanol‐derived films were prepared at 350 and 450 °C under otherwise identical conditions to assess the effect of deposition temperature.

For the growth of SnP/Sn_4_P_3_, the precursor solution consisted of 0.12 g of PH_2_CONHC_6_H_5_ (7.84 × 10^−4^ mol) and 0.10 g of tin(IV) acetate (2.17 × 10^−4^ mol) in 15 mL of dry chloroform, with the deposition temperature set at 350 °C. Additional films using chloroform as the solvent were prepared at 300 and 400 °C under otherwise identical conditions. All other parameters were unchanged.

### 4.3 Structural Characterization

NMR spectroscopy (Bruker AVIIIHD 400 MHz) was employed to analyze the PH_2_CONHC_6_H_5_ precursor. The thin films were characterized by XRD (grazing incidence, incidence angle = 1˚) on a Rigaku SmartLab using parallel Cu K_α_ X‐rays (*λ* = 1.5418 Å) and a Hypix detector in 1D mode. Scanning electron microscopy used a Zeiss Sigma 500 VP operating at 10 kV with an Oxford Ultim 170 EDS detector.

### 4.4 Sodium Half‐Cell Assembly and Electrochemical Measurements

To prepare binder‐free electrodes (mass loading of 0.16 mg cm^−2^), 11 mm disks were punched from the tin phosphide thin films on titanium foil and dried at 80 °C under vacuum overnight. Two‐electrode Swagelok cells were assembled using 12 mm commercial sodium disks (Suzhou Fosai, 99.7% purity) as the counter electrode inside an argon‐filled glovebox (<0.1 ppm O_2_ and H_2_O). A 1 mol dm^−3^ solution of NaPF_6_ in ethylene carbonate (EC)/diethyl carbonate (DEC) (1:1 vol ratio) with 2 wt% fluoroethylene carbonate (FEC), supplied by Suzhou Fosai Ltd., was used as the electrolyte. The electrochemical performance was tested with a Biologic BCS‐805 battery cycler at 25 °C. The cells were galvanostatically charged and discharged in a voltage range of 0.005 to 3 V at 200 mA g^−1^. Cyclic voltammetry (CV) curves were recorded from 0.1 to 3 V at a scan rate of 0.1 mV s^−1^. EIS were collected with a frequency range of 10 MHz to 10 mHz and a perturbation amplitude of 10 mV.

### 4.5 Ex Situ XRD and XAS Measurements

Ex situ methods were applied to probe the phase compositions of SnP/Sn after treatment in sodium half‐cells. The cells were charged and/or discharged at 150 mA g^−1^ to chosen potentials and then disassembled in the argon glovebox. After rinsing with dry dimethyl carbonate (DMC), the electrodes were placed into a sealed sample holder for XRD measurements or sealed in aluminized polybags for collecting XAS data. XANES and EXAFS data were collected at the Sn K‐edge (20.9 KeV) and P K‐edge (2145 eV) using fluorescence mode at the B18 beamline of Diamond Light Source. Sn foil, SnO_2_, and Na_15_Sn_4_, and red P were used as edge position references. All the XANES and EXAFS data were aligned, normalized, and merged in Athena. EXAFS results were processed in Artemis using FEFF6 theoretical models for β‐Sn metal, SnP, and Na_15_Sn_4_ paths. In EXAFS fitting, each path donates a specific photoelectron scattering pathway, which reflects the local environment of the absorbing atom.

## Funding

This work was supported by Diamond Light Source (SP38406), and Engineering and Physical Sciences Research Council (EP/K00509X/1, EP/K009877/1, and EP/V007629/1).

## Conflicts of Interest

The authors declare no conflicts of interest.

## Supporting information

Supplementary Material

## Data Availability

The data that support the findings of this study are openly available in the University of Southampton institutional repository at https://doi.org/10.5258/SOTON/D4047.
