# Supplementary material for: Aerosol‐Assisted Chemical Vapor Deposition of Mixed Phase Tin Phosphide Thin Films and Their Anode Performance for Sodium‐Ion Batteries
Source: ChemSusChem. 2026 Nov 5;19(21):e71205. doi: 10.1002/cssc.71205 (PMC13738049; doi:10.1002/cssc.71205)
Supplement: Supplementary file 1 [file CSSC-19-e71205-s001.pdf]

Aerosol-assisted chemical vapour deposition of mixed phase tin phosphide thin films, and their anode performance for sodium-ion batteries

Kaiqing Fan,[a] Xiang Lian,[a] Kangping Ma,[a] Veronica Celorrio,[b] Geoffrey Hyett [a] and Andrew L. Hector [a],*

[a] Kaiqing Fan, Xiang Lian, Kangping Ma, Prof Geoffrey Hyett, Prof Andrew L. Hector
School of Chemistry and Chemical Engineering
University of Southampton
Highfield, Southampton SO17 1BJ, UK
E-mail: Andrew Hector (A.L.Hector@soton.ac.uk)

[b] Dr Veronica Celorrio
Diamond Light Source
Harwell Science & Innovation Campus, Didcot, OX11 0DE, UK

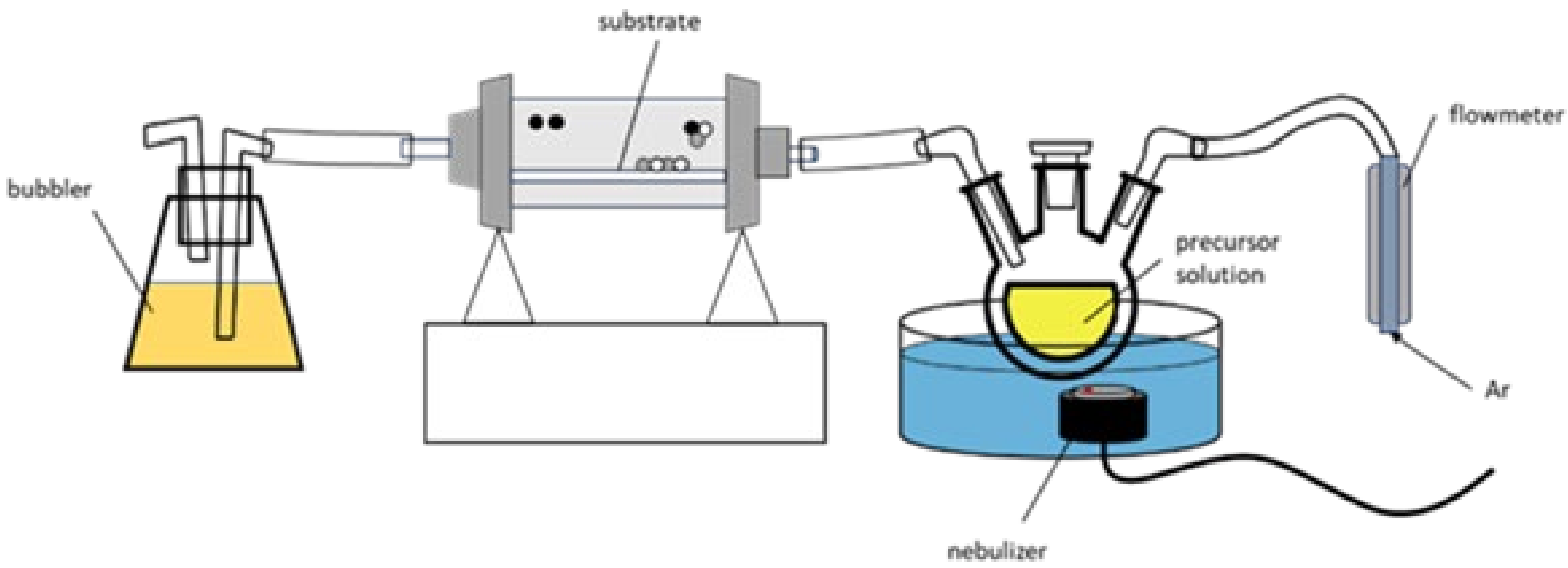

Figure S1. Schematic diagram of the AACVD setup used for the deposition of tin phosphide.

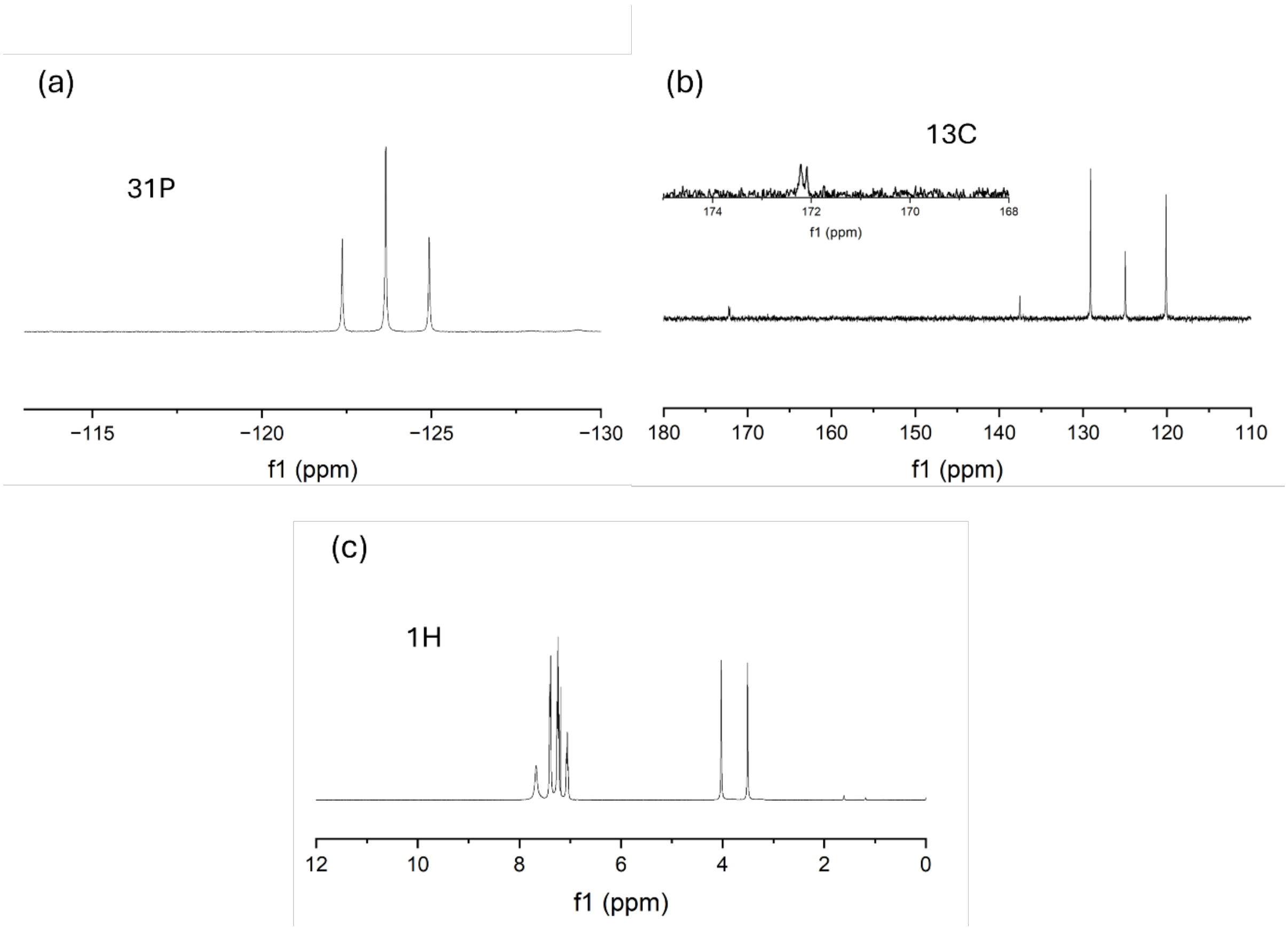

Figure S2. (a) ^{31}P, (b) ^{13}C, and (c) ^{1}H NMR spectrum of $PH_2CONHC_6H_5$.

Table S1 Synthesis parameters, crystalline phases, active material loadings, and electrochemical performances of all the deposited films.

Sample number	P/Sn ratio	susbatrate temperature	gas flow (sccm)	solvent	concentration	phase	active material mass (mg)	Electrochemical performance (200 mA/g after 100 cycles)
Me-350		350			0.1 in 15 ml	SnP/Sn	0.1	failed after 24 cycles
Me-425 (SnP/Sn)		425		methanol	0.12 g in 15 ml	SnP/Sn	0.15	507
Me-425	3:1	450	180		0.12 g in 15 ml	SnP/Sn	0.18	428
Ch-300		300			0.12 in 15 ml	Sn4P3	0.08	378
Ch-350(SnP/Sn_4P_3)		350		chloroform	0.12 in 15ml	SnP/Sn_4P_3	0.1	531
Ch-400		400			0.12 in 15 ml	SnP/Sn_4P_3	0.1	failed after 62 cycles

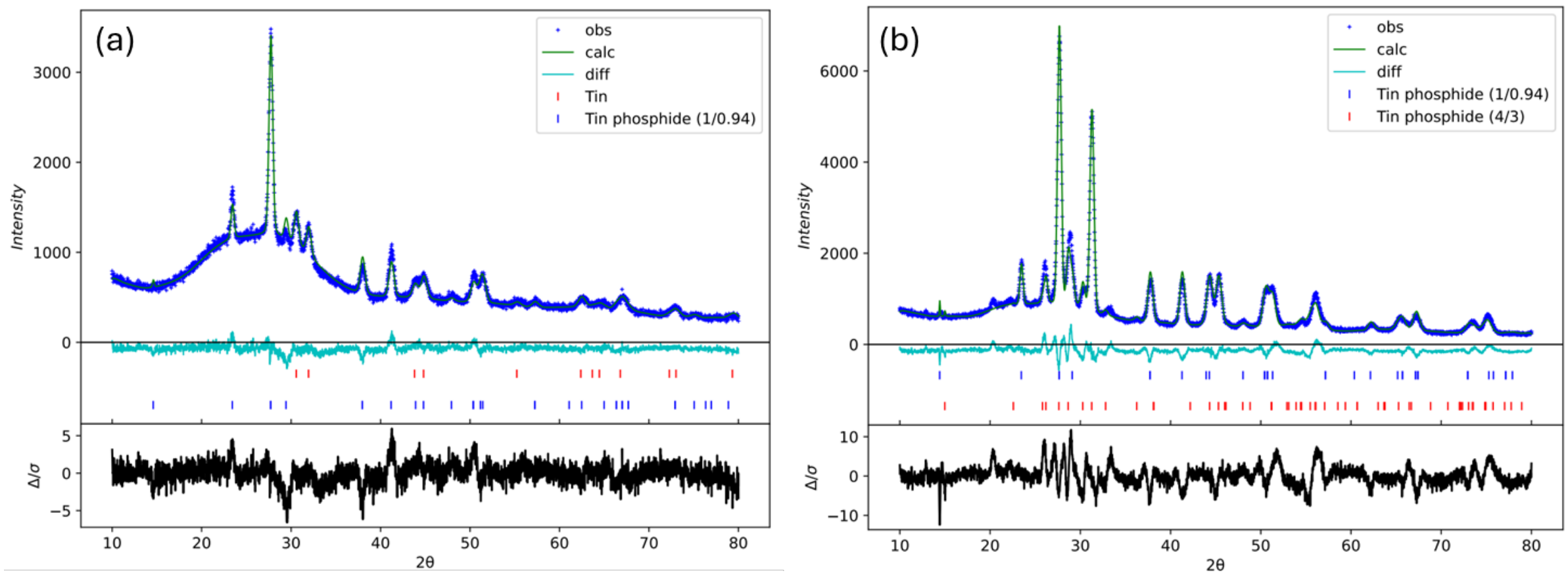

Figure S3. Rietveld fit of the XRD pattern of (a) SnP/Sn and (b) SnP/Sn_4P_3. The data points and calculated results are in blue and green crosses. The red and blue marks indicate the reflection positions of SnP P-3m1 (blue), tin $I4_1/amd$ (red in a), and Sn_4P_3 R-3mH (red in b).

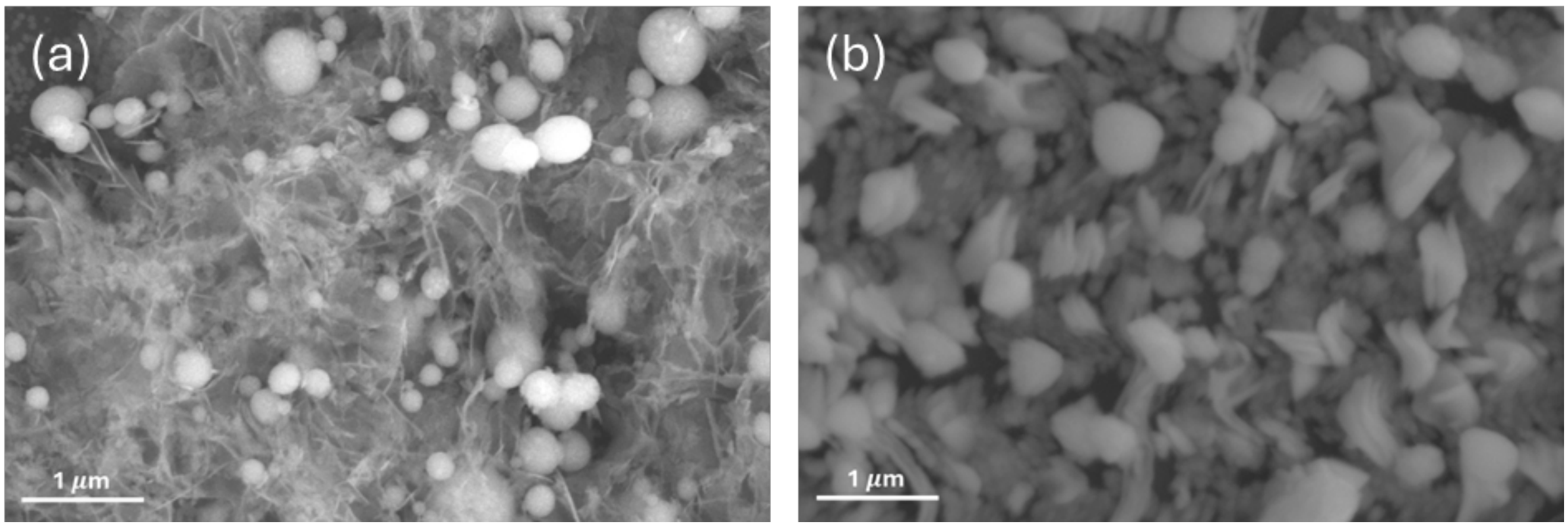

Figure S4. SEM images of (a) SnP/Sn grown under 425 ℃ and (b) SnP/Sn_4P_3 grown under 350 ℃ on glass substrates.

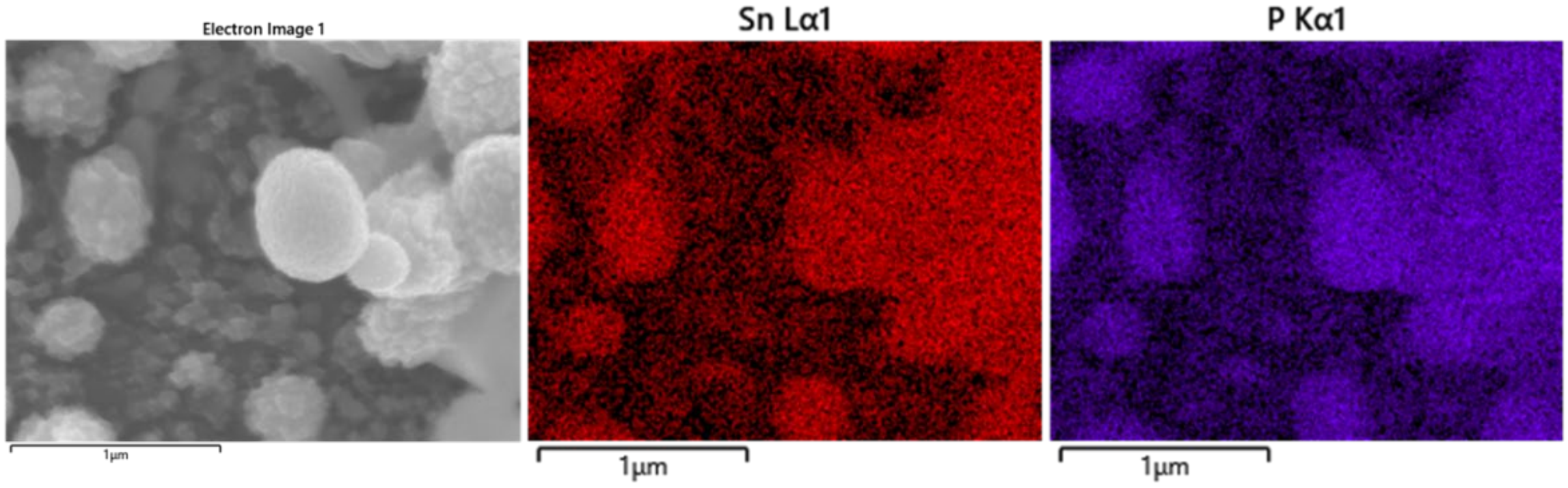

Figure S5. EDX element mapping of Sn and P for SnP/Sn_4P_3.

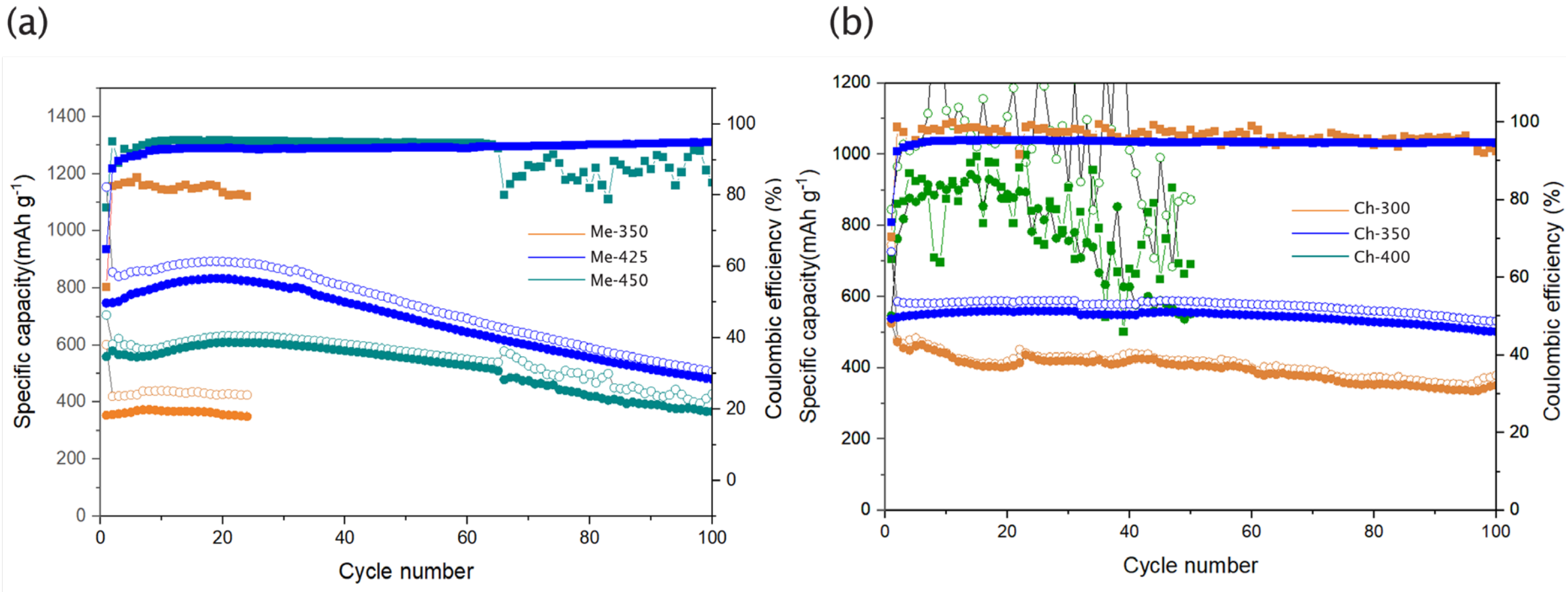

Figure S6. Cyclic performances of films made from (a) methanol and (b) chloroform at 200 mA g^{-1}.

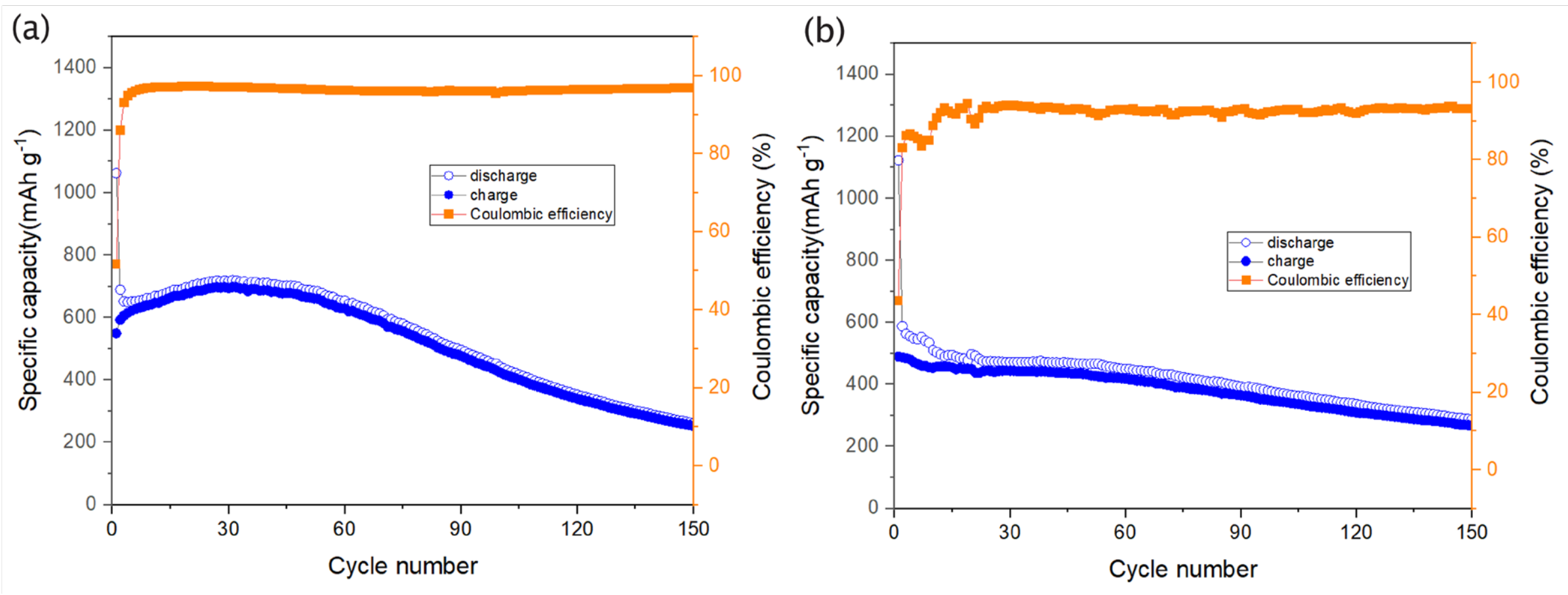

Figure S7. Cyclic performances of (a) SnP/Sn and (b) SnP/Sn_4P_3 at 600 mA g^{-1}.

Table S2. Estimated oxidation states of SnP/Sn during reduction and oxidation based on K-edge energies of samples and reference standards.

μ (E)	Process	K-edge (eV)	Oxidation state
	Pristine	29202.6	+2.49
1.05 V	Reduction	29201.7	+1.76
0.30 V		29201.1	+1.21
0.005 V		29200.6	+0.70
3.0 V 1st	Oxidation	29201.9	+1.93
3.0 V 2nd		29202.3	+2.25

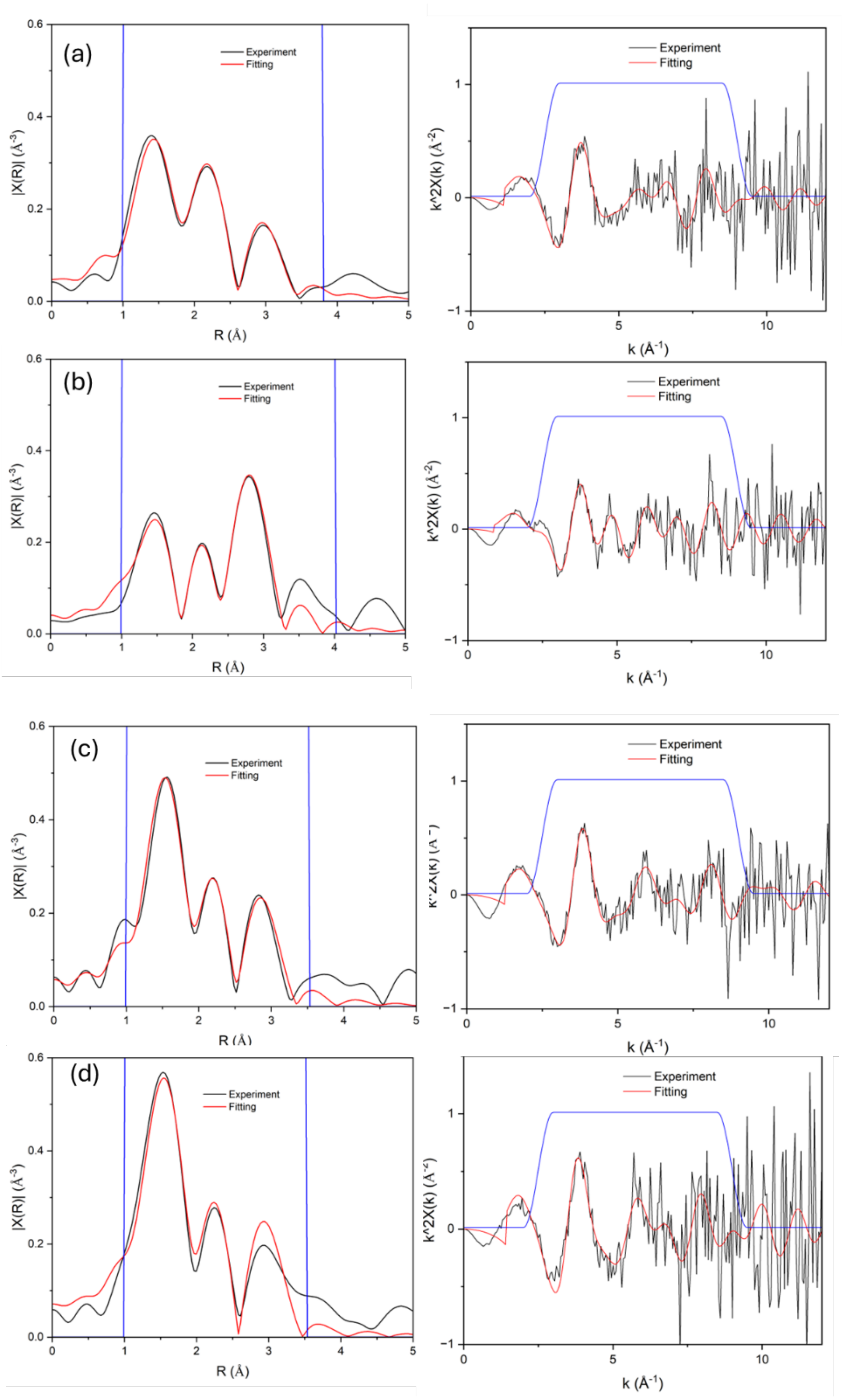

Figure S8. EXAFS fitting in k-space and R-space for (a) pristine, (b) first sodiated, (c) first desodiated, and (d) second desodiated.

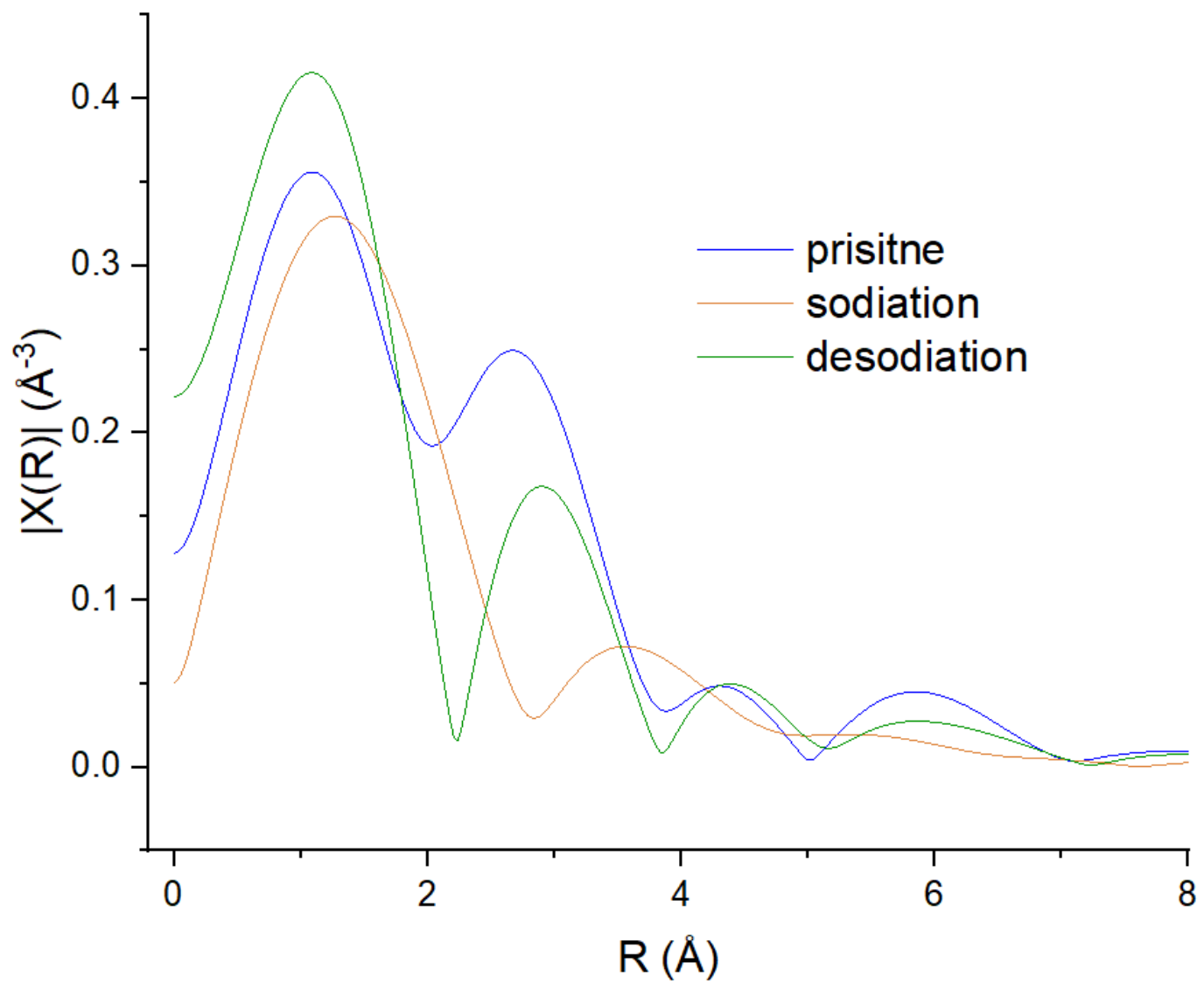

Figure S9.P K-edge |χ(R)| profiles of ex-situ EXAFS signals of SnP/Sn at OCV, first sodiation, and firsr desodaition.

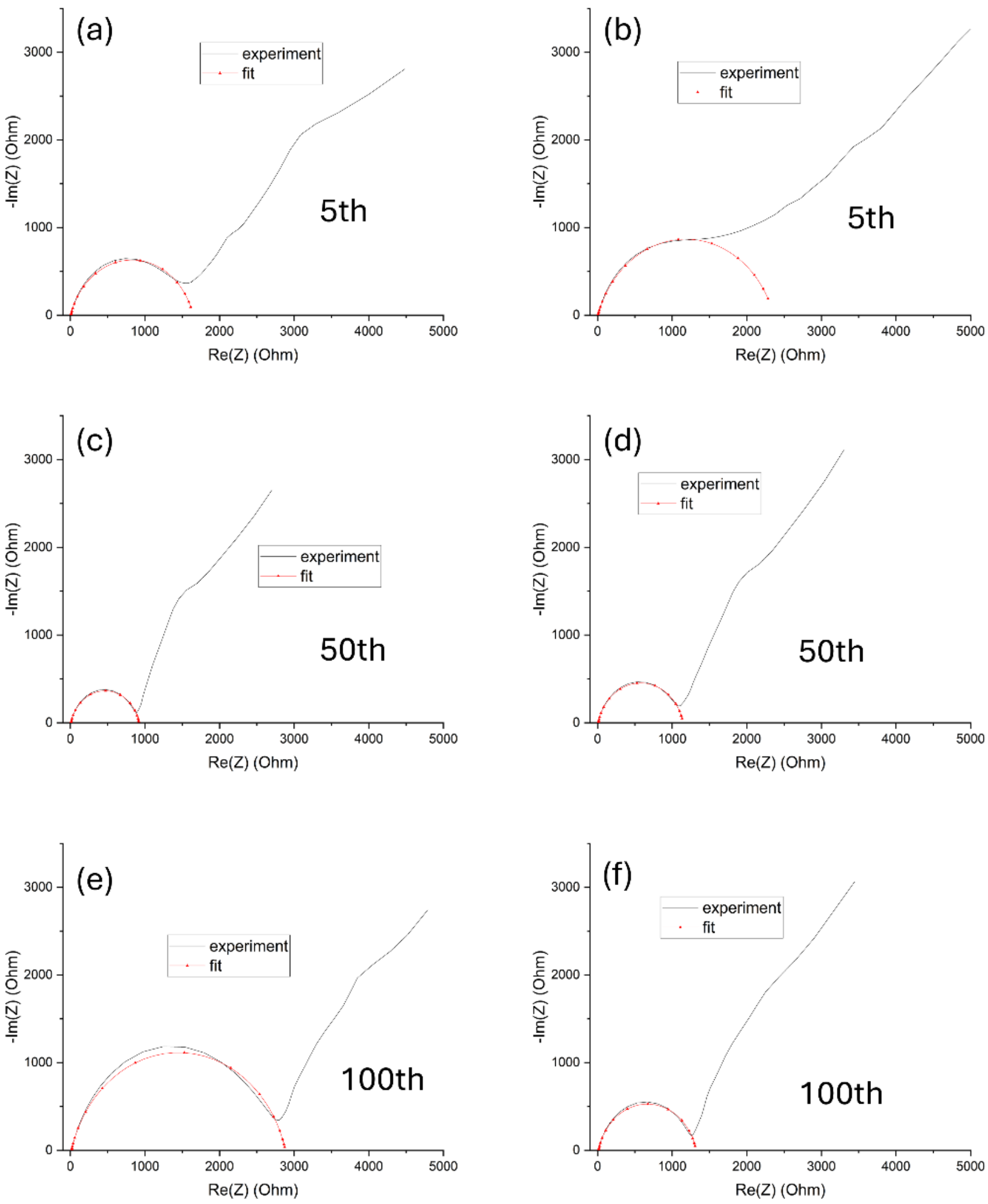

Figure S10. Nyquist plots of (a, c, e) SnP/Sn and (b, d, f) SnP/Sn_4P_3 at 10th, 60th and 100th cycles, and the fitted curves in high-frequency region.

Table S3. Calculated interfacial resistance (including the SEI impedance and the charge-transfer resistance) of SnP/Sn_4P_3.

Sample	Cycle number	Interfacial resistance (R_{int}, Ω)
Sn/SnP	5	1637
	50	910
	100	2866
SnP/ Sn_4P_3	5	2356
	50	1136
	100	1305
